# The contribution of small-scale rural irrigation schemes towards food security of smallholder farmers in Zimbabwe

**DOI:** 10.4102/jamba.v11i1.674

**Published:** 2019-10-09

**Authors:** Smart Mhembwe, Newman Chiunya, Ernest Dube

**Affiliations:** 1 Department of Development Studies, Zimbabwe Ezekiel Guti University, Bindura, Zimbabwe; 2 Department of Development Studies, Zimbabwe Open University, Gweru, Zimbabwe

**Keywords:** food security, irrigation schemes, livelihoods, irrigation rehabilitation

## Abstract

Smallholder farmers across Zimbabwe have been facing a problem of food insecurity because of climate-induced droughts and lack of effective use of irrigation schemes. Rainfall patterns in the country have become more unpredictable and inconsistent with the traditional farming seasons. Faced with such challenges, many smallholder farmers in Shurugwi district in the Midlands province of Zimbabwe adopted small-scale irrigation schemes to improve food security. The principal objectives of this study were to examine the status of the irrigation schemes in the district; analyse the need to rehabilitate small-scale irrigation schemes; assess the initiatives towards the revival of irrigation schemes; establish the benefits that can accrue to smallholder farmers from small-scale irrigation schemes and discuss challenges faced by smallholder farmers in the running of small-scale irrigation schemes in rural areas. This qualitative study employed literature and interviews to obtain data from 40 purposively selected participants. The direct observation method was used to compliment the interviews. The findings of the study were that small-scale rural irrigation schemes have the capacity to significantly transform the lives of rural farmers through earning increased reliable income from farming if institutional and capacity issues of the farmers are addressed. Furthermore, the study found that small-scale irrigation schemes can also be a panacea to food security challenges mainly faced by rural households. As such, the article concluded that irrigation schemes are fortress and antidote to the effects of climate change. The study calls for capacity promotion on technical skills for the farmers, the establishment of many new irrigation schemes and the rehabilitation of the existing small-scale irrigation schemes in the country as well as calling on the farmers to adopt climate-smart irrigation.

## Introduction

The adoption of irrigation schemes is an essential policy to eradicate poverty and improve food security for many governments across Africa. Such policy option greatly impacts on the livelihoods of rural communities where agriculture is the bedrock of their lives (Gebrehiwot, Mesfin & Nyseen 2015). Furthermore, Muzerengi and Mapuranga (2017) note that small-scale irrigation schemes in many less developed countries, particularly in Africa, were initiated mainly to boost agricultural production. In addition, these schemes were meant to reduce farmers’ dependency on unpredictable rainfall that characterises African climatic conditions (Muzerengi & Mapuranga 2017). Thus, the revival of small-scale irrigation schemes for farmers facilitates economic transactions, and improves livelihoods, wealth and infrastructure for communities (Christine et al. 2008). As such, one can note that the rehabilitation of irrigation schemes in Africa promotes food security and improves the standard of living of the rural people (Hussain & Hanjra 2014). This further shows a multifaceted role of irrigation schemes in contributing towards food security. In Zimbabwe, small-scale irrigation systems are critical common property resources that are needed to increase crop water supply in the fields to sustain livelihoods, particularly in semi-arid regions. Moyo et al. (2017) reflect that in semi-arid regions, rainfall is erratic and unreliable for dry farming. It is however, under such circumstances, that irrigation schemes become very critical for the success of agriculture, with approximately 80% of agricultural land in Zimbabwe lying in arid and semi-arid regions (Moyo et al. 2017). This may be the reason why the largest number of food-insecure households in Zimbabwe are mostly found in ecological regions 4 and 5. Communal households in these regions find it difficult to grow food on dry land, given the prevailing negative factors such as high temperatures, high incidences of agricultural failures and low rainfall.

In addition, it is argued that irrigation schemes for smallholder farmers in Zimbabwe are a mitigation measure, especially against droughts and the mid-season dry spells where crops severely suffer from moisture stress (Chazovachii 2012). Thus, irrigation can allow farmers to intensify crop production throughout the year. This is the major aim of irrigation development and rehabilitation of small-scale irrigation schemes, which might guarantee food security. It has further been noted that Zimbabwe basically has three broad types of small-scale irrigation schemes, namely, those managed by the government, those managed by farmers as individuals and those jointly managed by communities (FAO 1996).

Chitora irrigation scheme in Mutuko district is one example of an irrigation scheme in Zimbabwe that is playing a pivotal role in the reduction of food insecurity, malnutrition and poverty, as well as contributing towards economic empowerment of the local people (Chikwati 2018). This scheme was funded by the Government of Zimbabwe and Danish International Development Agency (DANIDA). It is important to note that DANIDA is a long-time main actor in the funding of the small-scale irrigation schemes around the country (FAO 1997). In Zimbabwe, several small-scale irrigation schemes, especially in the rural areas such as Mutambara irrigation scheme in Chimanimani, Fuve-Panganai irrigation scheme in Zaka, Rusike and Mushandike irrigation schemes in Masvingo, have been shifting over the years from the traditional modes of irrigation to modern modes of irrigation (IFAD 2006). The traditional modes of irrigation normally make use of shallow wells, ponds and spring water, whilst the modern modes of irrigation are characterised by improved physical infrastructure, including the use of water pump engines, sprinklers and overhead irrigation equipment.

Small-scale irrigation schemes in Zimbabwe have for years been premised on the cycle of build–operate–rehabilitate. This kind of operation is heavily dependent on donor funding and support for sustenance. Thus, where there is lack of donor support, several irrigation schemes in the country would probably cease to function properly (IFAD 2006). However, the observation highlighted above best suits irrigation schemes in Matabeleland regions, which basically lack knowledge and finances to maintain irrigation equipment without external support (CESVI 2017). This has led to most of the rehabilitation of the irrigation schemes in the country to primarily focus on the development and repairs of physical infrastructure. This would be done, without corresponding investments in farmers’ institutional development, production activities and market linkages.

The rehabilitation of irrigation schemes in Zimbabwe has largely been managed by the Department of Irrigation under the then Ministry of Agriculture, Mechanisation and Irrigation Development (IFAD 2016). Furthermore, apart from the current Ministry of Lands, Agriculture and Rural Resettlement, there are other stakeholders that have been helping in the rehabilitation programme of small-scale rural irrigation schemes. For example, the Guyu and Chelesa irrigation schemes in Gwanda were rehabilitated by the Gwanda Community Share Ownership Trust to empower local people in terms of food production (Tshuma 2015).

Shurugwi district in the Midlands province is also one district that has been struggling for decades to be food secure, with its population depending mostly on rain-fed agriculture without substantive irrigation. This is because the district lies in the semi-arid region that receives an average annual rainfall of between 650 mm and 800 mm (Matsa & Masimbiti 2014). However, of late, the district managed to evolve from traditional small-scale irrigations, such as the use of shallow wells, ponds and spring water, to modern irrigation facilities with permanent structures and improved water control systems. Currently, there are a few modern small-scale irrigation schemes in Shurugwi district that have been developed and rehabilitated with the help of various stakeholders. These stakeholders include non-governmental organisations (NGOs) such as Linkages for the Economic Advancement of the Disadvantaged (LEAD); Ministry of Lands, Agriculture and Rural Resettlement; Tongogara Rural district Council; Zimbabwe National Water Authority and Zimbabwe Electricity Supply Authority, amongst others. All these stakeholders have been working with local communities as a way of helping smallholder farmers to improve their livelihoods through farming. The following are some of the small-scale irrigation schemes, which undertook some rehabilitation work to increase on productivity: Gutsaruzhinji irrigation scheme in ward 19, Zananda irrigation scheme in ward 7, Gonye irrigation scheme in ward 11, Senamwe irrigation scheme in ward 14 and Ruchanyu irrigation scheme in ward 20. These schemes play a pivotal role in promoting food security and empowerment of the rural people.

Thus, the central focus of the study was on the role played by small-scale irrigation schemes as a way of boosting food productivity and reducing poverty amongst smallholder farmers in Shurugwi district. The principal objectives of the study were to examine the status of the irrigation schemes in the district; analyse the need to rehabilitate small-scale irrigation schemes; assess the initiatives towards the revival of irrigation schemes; establish the benefits that can accrue to smallholder farmers from small-scale irrigation schemes and discuss challenges faced by smallholder farmers in the running of small-scale irrigation schemes in rural areas.

### Context of the problem in Shurugwi district

Irrigation schemes for smallholder farmers are essential policy options chosen by many governments in Africa with the intention to eradicate poverty and improve food security for their citizens (Gebrehiwot et al. 2015). Thus, small-scale irrigation schemes have a great impact on the livelihoods of rural communities across Africa, where agriculture is considered the bedrock of people’s lives. However, in Shurugwi district of Zimbabwe, whose greater part falls under agro-ecological region 4, the district continues to face low agricultural productivity because of low rainfall patterns and underutilisation of irrigable farming land found nearer to the water bodies. Considering the above, the Government of Zimbabwe, in partnership with other stakeholders, has tried to rehabilitate some of the small-scale irrigation schemes for smallholder farmers in the district with no success because of limited resources. The district has, for many years, been failing to ensure food security to its population because of erratic rainfall and little contribution of irrigation schemes. Thus, if the smallholder farmers in the district are not given external support to rehabilitate their small-scale irrigation schemes, many households would continue to experience high levels of poverty and hunger because of food insecurity.

### Review of related literature on food security and small-scale rural irrigation schemes

In the quest to unpack the role played by small-scale rural irrigation schemes towards food security of smallholder farmers in Zimbabwe, this section presents an extensive literature review. The section is divided into different sub-headings and pursued an understanding of the nexus between small-scale rural irrigation schemes and food security, as well as revealing the major challenges faced by such small-scale farmers. The literature review was done using the following sub-headings to promote clarity: the framework of the study; understanding food security; development of small-scale irrigation in Africa; small-scale irrigation schemes and food security for rural farmers; and challenges faced by small-scale irrigation schemes in Zimbabwe.

### The framework of the study: The Sustainable Development Goals

This study was conducted within the framework of the Sustainable Development Goals (SDGs), with specific focus on SDG1 that seeks to end poverty in all forms, and SDG2 that aims to end hunger, achieve food security and improved nutrition and promote sustainable agriculture (United Nations Development Programme [UNDP] 2015). On 01 January 2016, the United Nations’ 17 SDGs took effect, launching the countdown towards the achievement of 169 targets by 2030, and many of these ambitious targets are deeply significant for agriculture (OECD 2016). As such, within this framework, the authors argue that the role of irrigation is to end extreme poverty in all forms in human communities, as well as ending hunger through agricultural activities that improve food security and nutrition of households and communities. Before discussing in detail the relevance of the SDGs, it is of paramount importance to give a brief outline of the two goals informing this study (UNDP 2015):

- *Goal 1. No poverty:* End extreme poverty in all forms by 2030.
- *Goal 2. Zero hunger:* End hunger, achieve food security and improved nutrition and promote sustainable agriculture.

As can be observed, the aims of first two SDGs (Goal 1 and Goal 2) are to end extreme poverty in all forms and to end hunger, achieve food security and improved nutrition and promote sustainable agriculture. These goals make the SDGs a suitable framework for this study, which seeks to promote agriculture through improved irrigation systems. When irrigation systems are improved, food security of households and communities is likely to improve as well. The United Nations (2013) states that both these goals highlight the basic need of feeding the world’s population, which is expected to grow to 9.6 billion by 2050. The authors argue that the adoption of irrigation schemes by small-scale farmers can be a major contribution towards feeding the world’s population. It is also important to note that many of the poor populations constitute subsistence farmers in the rural areas, where approximately 70% of the developing world’s 1.4 billion extremely poor people live (Setboonsarng & Gregorio 2017). Therefore, if the livelihoods of small-scale irrigation farmers are improved, poverty and hunger would be a thing of the past. SDG1 is relevant in this study as agriculture is a key antipoverty strategy particularly in rural areas that can provide rural employment, lower input costs for small-scale farmers and raise their incomes (Setboonsarng & Gregorio 2017). As far as SDG2 is concerned, the use of irrigation schemes allows diversified cropping systems, reducing the risk of income losses associated with seasonal variations and crop failures (FAO 2002) that improves food security. Therefore, if the livelihoods of small-scale irrigation farmers are improved, it will contribute the eradication of poverty and hunger.

### Understanding food security

The concept of food security can be looked at from different angles by different scholars. According to FAO (1996), food security refers to as a situation whereby all people always have physical, social and economic access to enough, safe and nutritious food that meets their dietary needs and food preferences for an active and healthy life. Food security is taken to be a situation whereby all individuals in a population can produce and procure enough food for an active and healthy life (Rukuni, Mudimu & Jayne 1990). In addition, food security can also be looked at as the amount of food that is present in a country through all forms of domestic production such as imports, food stocks and food aid (Muzerengi & Mapuranga 2017). The above definitions show that food security can best be understood by looking at its four dimensions, namely, availability, access, utilisation and stability. The dimension of availability refers to a situation where households have a better and more sustainable availability of food through either production or buying of the food stuff (Christine et al. 2008). The second dimension of food access refers to the ability of households to acquire food through production, purchases in the market from income earned from transfers (Nhundu & Mushunje 2012). FAO (1996) further indicates that the third dimension of food utilisation places much emphasis on the ability by households to have a balanced diet of the food that is nutritious, whereas the last dimension of stability speaks to a situation where food is more solid and offers constant food supply throughout the year especially during periods of stresses and shocks and in terms of seasonality. This, however, shows that when talking of food security, one will be referring to a situation whereby households have access to the available food that meet their dietary needs at all the times.

### Development of small-scale irrigation in Africa

The history of irrigation is very old as it can be traced in the ancient times, as far as 4 BC, through archaeological evidence where remains of irrigation canals were found in the Zana valley of the Andes Mountain in Peru (Kang 1993). Furthermore, archaeological investigations have also provided evidence of irrigation in Mesopotamia, Ancient Egypt and Iran, which indicate that the practice dates back 6 BC. It has also been argued that irrigation farming is an ancient practice with a long history that can also be traced back in countries such as Ethiopia where the history of water harvesting started as early as the pre-Axumite period in 560 BC, when rain water was harvested and stored in ponds for agriculture and water supply purposes during the ancient period (Gebrehiwot et al. 2015). Since then, the practice of irrigation has been evolving over time from traditional modes of irrigation to modern modes. However, the evolution of the irrigation modes calls for the continuous rehabilitation of irrigation schemes to meet the demands of the farmers and the people at large in the present-day century.

In Ghana, the agricultural sector is considered vulnerable mainly because it relies more on rain-fed agriculture, a situation that often leads crops to be affected by mid-season dry spells resulting in wide spread crop failures (Namara et al. 2011). Thus, the rehabilitation of irrigation schemes in Ghana’s rural communities has been observed to be the mainstay in achieving food security, poverty reduction and rural employment (Namara et al. 2011). It has been further observed that in countries such as Ethiopia, the amount, frequency and distribution of rainfall, which is the source of water for crop production, are becoming more uneven and inadequate because of global warming that results in climate change (Namara et al. 2011). It is under such circumstances that irrigation farming would enhance successful crop growing and stabilise crop yields. Thus, one can note that irrigation is required in places that have uncertain and uneven distribution of rainfall. That is in most drought-prone areas, successful crop production is only possible with the support of irrigation.

In addition, Gebrehiwot et al. (2015) also note that agricultural production in Ethiopia used to rely mainly on rain-fed that was more often erratic and insufficient. Hence, the country has been experiencing frequent agricultural production failures all because agriculture was heavily affected by variability of rainfall and drought. Thus, given the above scenario, the government of Ethiopia paid special attention to enhance its agricultural production through the promotion of small-scale irrigation schemes mainly because most of low productivity areas had large quantities of water resources that were largely untapped (Gebrehiwot et al. 2015). It has been further observed that Ethiopia is one country that has managed to revive its irrigation farming especially for smallholder farmers as a way of enhancing rural development and ensuring food security (Christine et al. 2008). This, however, is an indication that irrigation farming has the potential to stabilise agricultural production and reduce the negative impacts of sporadic and insufficient rainfall on the farmers.

FAO (2005) also shows that the Zambian government has implemented irrigation schemes as a way of addressing the challenges of drought that the country has been facing, which often led to massive food insecurity to the nation. That is, in the late 1960s and 1970s, the Government of Zambia developed and managed smallholder irrigation schemes through the Projects Division of the then Ministry of Rural Development. Amongst the prominent schemes that were developed included the Buleya-Malima, Chapula and Mulumbi, amongst others. However, because of the top-down approach to the management of these irrigation schemes, most of them did not perform to expectations with the majority completely ceasing operations. The Zambian government later came up with a national irrigation policy that focused on rehabilitation of the abandoned schemes where expertise assistance and irrigation equipment were provided to the farmers. Furthermore, the management responsibility for operations and maintenance of the irrigation systems were given to the beneficiaries as a way of inculcating a sense of ownership to enable communities to take good care of the irrigation equipment (Aquastat 2005).

In Malawi, because of the intensification of climate change that results in erratic and unpredictable rains, Oxfam has been working with poor communities to improve agricultural productivity and livelihoods through the development and rehabilitation of community-based irrigation schemes (Oxfam 2011). A good case in point is that of the Mnemba irrigation scheme, which was established in 2004 with the assistance of Oxfam and since then, the scheme has been acting as a source of livelihood for several families where yields were transformed leading to high-volume harvests. Thus, food security for most poor Malawians was better achieved through initiatives in investing in the productivity of small-scale irrigation farming (Oxfam 2011).

Irrigation is also critical to the South African economy where agriculture, forestry and fisheries contributed about 2.6% of South Africa’s Gross Domestic Product, with irrigated agriculture contributing over 30% of the gross value of the country’s crop production (Department of Agriculture [DoA] 2007). Over the past years, irrigated agriculture in South Africa has undergone several changes in terms of switching from grains, fodder and other similar crops to high value horticulture and cash crops coupled with the intensification of production. It has been further observed that irrigation plays an important role in the South African fruit industry that is ranked amongst the most important export commodities, with about 90% of the country’s fruit and wine produced under irrigation (Nieuwoudt, Backeberg & Du Plessis 2004). Although vegetables are not an important export crop as the fruit and wine crops, but by producing 90% of the country’s vegetables, irrigation is essential for ensuring a healthy diet for the nation and contributes intensively to national food security (DoA 2007).

In Zimbabwe, the history of irrigation development for smaller holder farmers became prominent in the early 1980s when the new government partnered with several development agencies in establishing small-scale irrigation schemes mainly for the rural communities (Nhundu & Mushunje 2012). Thus, the Government of Zimbabwe in 1982 partnered with the European Micro Project Programme in funding the establishment of smallholder irrigation schemes across the country. Furthermore, it is indicated that this was necessitated by the fact that many black smallholder farmers were restricted to arid regions where the soils were poor and with little rainfall; hence, the schemes were developed as a famine relief strategy (Nhundu & Mushunje 2012). However, over the years, several such schemes stopped operations owning to the fact of dilapidated infrastructure and lack of capacity to maintain the equipment. The introduction of blue prints such as the Zimbabwe Agenda for Sustainable Socio-Economic Transformation (ZIMASSET) and the adoption of the policy on More Food for Africa are indications that the government realised the need to rehabilitate all the irrigation schemes. Such schemes were either those struggling with their operations owing to old equipment or those that had since stopped their operations because of several challenges. This was done as a way of enabling the nation to be food secure after realising the negative effects of climate change on agricultural production where, as a nation, severe food insecurity was experienced over a couple of years ago. The stance by government to rehabilitate small-scale irrigation schemes invited several stakeholders on board to come and assist smallholder farmers in trying to boost agricultural productivity so that households and communities become food secure.

### Small-scale irrigation schemes and food security for rural farmers

Faced with the effects of climate change that are contributing negatively to issues of food security, governments across Africa have adopted the practice of irrigation farming as a mitigation measure to issues of famines and hunger for their own citizens (Fleshman 2007). Thus, with the increase in urbanisation and the underlying problem of substantial poverty coupled with the negative impacts of global warming, many countries are battling to become food secure. It has been noted that agriculture productivity on the African continent is being heavily affected by variability of rainfall and drought where rainfall is becoming more erratic than ever (Gebrehiwot et al. 2015). Given such a scenario, farmers especially those in poor areas continue to suffer from chronic poverty and severe food insecurity because of being vulnerable to climate change and depending more on variable rainfall.

It has been further observed that irrigation farming lessens the risk of catastrophic damages to crops and people’s livelihoods (Waugh 2009). These damages include crop failure, loss of domestic and wild animals, and nutrients being lost in the soil and in extreme cases; this can also lead to the deaths of human beings because of hunger. This, however, shows the need for nations to move away from relying on rain-fed agriculture through adopting irrigation farming where farmers can easily regulate crop water requirement to increase productivity (Fleshman 2007). The available literature also shows that irrigation schemes for smallholder farmers help in relieving farmers from being dependent on rain-fed agriculture. Also, where water is available, farmers have the capacity to easily increase the size of their farmlands as a way of boosting productivity, thereby helping them to accumulate wealth through selling of surplus produce (Muzerengi & Mapuranga 2017). Furthermore, it has been noted that the wealth accumulated because of farming through irrigation will also act as a guarantee to food security in the event of crop failure and that irrigation farming helps in generating employment as well as encouraging farmers to produce an average of 2–3 times a year (Food and Agriculture Organisation [FAO] 2000). The development of smallholder irrigation schemes has been found increasing the potential for more production especially through countering the mid-season and periodic dry spells, thus households are able to grow crops more than once in a year (Nhundu & Mashunje 2012. This reflects that irrigation development for smallholder farmers helps in cushioning households in times of food insecurity and at the same time leading to various income-generating activities in the rural economy (FAO 2000). Furthermore, such income-generating activities will, as a result, lead to the creation of off farm employment where livelihoods are earned through selling the agricultural produce from the irrigated land (Muzerengi & Mapuranga 2017).

Gebrehiwot et al. (2015) also argue that there is a stronger linkage between irrigation development and poverty reduction. However, this linkage is strengthened through increased productivity, livelihood diversification as well as employment opportunities and income generation from irrigation farming. The sentiments raised above are in line with the observations by Lankford (2003) who argues that irrigation promotes a more secure and increased crop productivity, improved planning and timing of start of the cropping season and extended harvest season, and above all, more number of jobs and enhanced income. Oxfam (2011) also indicates that the development of irrigation for smallholder farmers does not only offer them the potential to move to all year round cropping something that enables them to generate higher annual yields from a single plot, but also allows these farmers to diversify and plant alternative cash crops to earn an income.

In addition to the above, smallholder irrigation systems are viewed as critical common property resources that are needed to increase crop water supply and sustain livelihoods in semi-arid regions (FAO & WWC 2015). Improving agriculture and enhancing productivity through small-scale irrigation is one of the key strategies for alleviating poverty and improving the livelihoods of smallholder farmers. Most of the poor depend directly or indirectly on agriculture (Mutiro & Lautze 2015). Thus, one of the main targets of developing irrigation systems across nations is to capacitate rural small-scale farmers to produce enough food for both consumption and sell (Mengistu 2003). Thus, irrigation for smallholder farmers is regarded as a powerful factor in providing food security, protection against adverse drought conditions, increased prospects for employment and stable income and greater opportunity for multiple cropping and crop diversification.

### Challenges faced by small-scale irrigation schemes in Zimbabwe

On a global perspective, smallholder irrigation schemes are evidenced in several countries as a strategy to curb food insecurity in many rural areas. It has been put forward that many smallholder irrigation schemes though profitable face several obstacles in trying to achieve food security as in most cases these schemes are too small for them to have economies of scale and consequently falling into the trap of low levels of technology (Denison & Manona 2006). They also lack access to proper institutions and organisations that can provide the necessary assistance for them to be more viable. For example, the situation can be aggravated by irrigation systems that are uneconomic by design where such schemes experience high production costs with little chance of cost recovery. In addition, it is also argued that several challenges such as poor marketing arrangements, limited access to water, lack of sense of ownership, problems of financial viability and issues of poor governance have befallen most small-scale irrigation schemes in Zimbabwe, and it is something that contributed to their dilapidation and vandalism of equipment (Rukuni et al. 1990). Thus, poor maintenance and lack of effective control over irrigation practices have resulted in the collapse of many irrigation systems across the globe over the years.

Vandalism has also played a negative role on small-scale irrigations leading to the reduction of their outcomes. Damages such as the destruction and breaking of sprinklers and agricultural machinery, breaking the concrete pipes protecting the risers, stealing of irrigation valves and fittings, stealing of the locks and the doors of pumping stations and avulsing and stealing drip irrigation pipes are some of the common threats faced by rural small-scale irrigation schemes (Nhundu & Mushunje 2010). For example, it has been noted that vandalism at one moment in time stalled the agricultural activities of Chelesa and Guyu irrigation scheme in Gwanda after several irrigation pipes were stolen by local villagers (Maodza 2012). Furthermore, vandalism of electricity transformers has been noted to be a common trend in Zimbabwe and this has contributed to power outages where schemes will go for weeks without electricity to power their water pump engines (Maodza).

The Government of Zimbabwe’s main objective for small-scale irrigation development is to guarantee food security through increased crop production (Chazovachii 2012). However, most of the schemes fell short of achieving this noble objective because of complex interrelated factors such as low technical capacity, poor institutional arrangements and uncoordinated market linkages and because of exorbitant charges on water by bodies such as Zimbabwe National Water Authority (ZINWA) (Mujere et al. 2011). Thus, in Zimbabwe, several small-scale irrigation schemes are characterised by low production, minimal contribution to the economy and inability to cover development and operations costs because of the above-mentioned challenges (Moyo et al. 2017).

### Description of the study area

**FIGURE 1:**
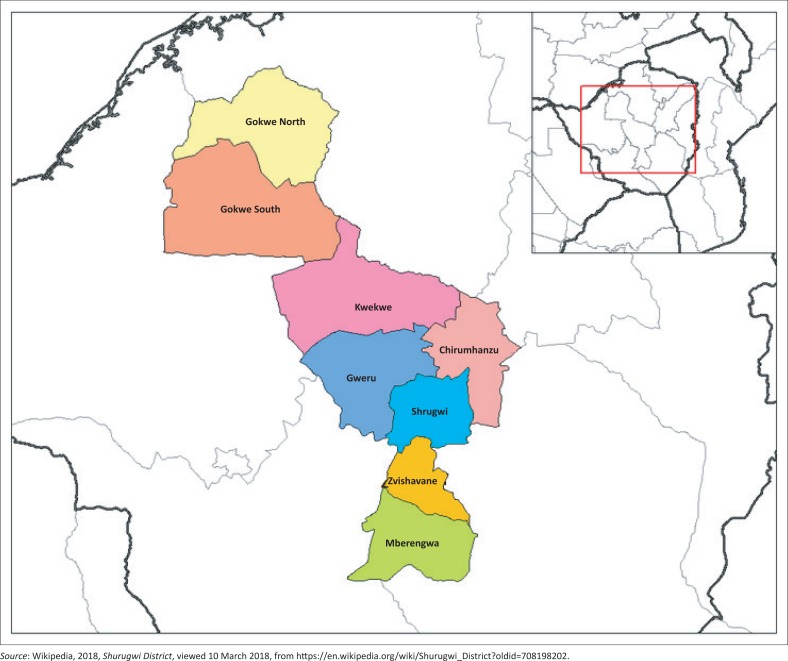
Map of Shurugwi district in the Midlands province.

The study site is Shurugwi district, which is one of the eight districts in the Midlands province of Zimbabwe (Figure 1). The district has an estimated population of 99 475 people (Zimstat 2012). It is categorised under agro-ecological region 3, which is characterised by warm temperatures, and an average annual precipitation of between 650 mm and 850 mm (Matsa & Masimbiti 2014). The major land uses in Shurugwi district include residential, agricultural, mining, crop and livestock farming, which are the dominant practices (Matsa & Muringaniza 2010). The district has a total of more than 10 small-scale irrigation schemes, and a total of about 250 ha of agricultural land have been put under irrigation. Farmers in the district draw water for irrigation purposes mainly from dams, rivers and boreholes.

## Research design

This study was based on a case study research design. Case studies focus on a contemporary phenomenon within real-life context (Magwa & Magwa 2015). Thus, the study focused on the contribution of small-scale irrigation schemes to food security. According to Corbin and Strauss (2015), qualitative approach allows researchers to collect and interpret data, with the researcher being part of the research process. Therefore, qualitative research approach was adopted to allow the researchers to use instruments that collect observed data. Furthermore, qualitative research allowed the respondents to narrate their experiences, opinions and beliefs about small-scale irrigation schemes. This type of approach is inductive, discursive and interpretive. The observation method added value to the study as the researchers managed to get first-hand information from the areas studied. In-depth interviews were also conducted in this study. The advantage with in-depth interviews is that they afford researches to gather more detailed information. The timeframe for data collection was 2 months and activities were carried out during dry season to observe the use of irrigation equipment.

## Sampling

A sample of 40 research participants was selected through the purposive sampling and this technique is a non-probability technique. The sample was arrived at through a saturation process. The 40 participants provided commensurate information needed for the research, to the extent that when any additional participant was engaged, no new and useful information could be extracted, but a mere repetition of what has already been collected. Dornyei (2007) states that in non-probability sampling technique qualifying and disqualifying criteria are used. In this study, the sample comprised 32 male and female farmers working in irrigation schemes in Shurugwi district, as well as eight government officials drawn from government departments in the district, namely, the Department of Agritex, Department of Mechanisation and Irrigation and the District Administrator’s office. Table 1 illustrates a breakdown of the respondents who constituted the sample according to their gender and level of education.

**TABLE 1:** Breakdown of respondents according to gender and level of education (*N* = 40).

Gender	Frequency	Percentages	Level of education			Totals
			Primary	Secondary	Tertiary	
Male	31	78	15	12	04	31
Female	9	22	5	4	0	9

**Totals**	**40**	**100**	**20**	**16**	**04**	**40**

From Table 1, it can be observed that both male and female respondents formed the sample for the study. The male respondents constituted 78% of the sample, whilst female respondents were 22%. This variation implies that farming work is a male-dominated activity in Zimbabwe. Furthermore, a breakdown of the level of education of the research participants is shown in Table 1. A total of 20 out 40 respondents had reached primary education, whilst 16 out of 40 and 4 out of 40 respondents had attained secondary and tertiary education, respectively. The level of education implies that more education, especially farming education, is needed to empower the small-scale farmers in Shurugwi district. Table 2 is an illustration of the types of crops grown by the small-scale farmers in the district.

**TABLE 2:** Types of crops grown by small-scale farmers in Shurugwi district (*N* = 32).

Type of crop	Frequency	Percentage
Maize	15	37.5
Cow peas	10	25.0
Sugar beans	10	25.0
Sugar cane	05	12.5

**Total**	**40**	**100**

The main types of crops grown by smallholder farmers in Shurugwi district include maize, cowpeas, sugar beans and sugar cane. According to the respondents, maize (37.5%) is most commonly grown crop, followed by cowpeas (25.0%) and sugar beans (25.0%), whilst sugar cane (12.5%) is the least common crop grown (Table 2). Researcher observations also revealed that maize was common with almost all pieces of land where irrigation was practised.

## Ethical considerations

This research observed all research ethics for protecting the respondents and the data collected.

## Results and discussion

### Status of the irrigation schemes in Shurugwi

Gutsaruzhinji and Ruchanyu irrigation schemes in Shurugwi were established during the first phase of land reform in Zimbabwe in the early 1980s. According to the respondents, Ruchanyu irrigation scheme is run by 85 smallholder farmers, whilst Gutsaruzhinji is managed by 42 smallholder farmers. The study further observed that Gutsaruzhinji irrigation scheme occupies 21 ha, whilst Ruchanyu irrigation scheme sits on 27 ha. It is striking to note that the farmers running the two schemes were drawn from local communities surrounding these schemes. The study also revealed that the composition of the farmers running Gutsaruzhnji and Ruchanyu schemes was largely dominated by elderly men (65%), followed by elderly women (25%) and youths (10%). This is an indication that rural communities are patriarchal in nature, considering that men are most of the people who own the land. There is little regard to the youths as they own a small portion of the land.

This study established from the respondents that over the years these irrigation schemes were heavily affected through vandalism of the irrigation equipment, as well as neglect by the farmers themselves because of lack of sense of ownership. The vandalism of irrigation equipment compromises food security because food production would be stagnant, whilst the vandalised equipment is being replaced. A good case in point was that of Gutsaruzhinji, which, respondents indicated, had its power disconnected by the electricity utility organisation for non-payment of electricity bills. As for Ruchanyu, the scheme had been suffering from serious disruptions in irrigation activities because of vandalism of the irrigation water pump engines. The researchers further established, through the respondents, that about 6 km of electricity cables feeding power to Ruchanyu irrigation scheme were stolen in 2012. Whilst vandalism and theft of agricultural equipment compromise food security, these acts are also against the objectives of the SDGs, which are the framework guiding this study. The objectives of the two main SDGs informing this study are to end extreme poverty in all forms (SDG 1) and to end hunger, achieve food security and improved nutrition and promote sustainable (SDG 2).

Furthermore, it was established through the interviews that a few irrigations schemes in the district are in areas where land is fertile and where water is in abundance. Thus, the study revealed that both Gutsaruzhinji and Ruchanyu schemes are located where soils are fertile and where water is in abundance as they all draw their water for irrigation from Mutevekwi River, which is a perennial river. The authors also argue that establishing irrigation schemes in fertile areas, and where water is in abundance, can be a panacea to improving food security especially in semi-arid regions.

However, of interest to note from the study is that, despite the location of these two irrigation schemes, where water is in abundance, the water sources themselves are not being fully utilised by the farmers. This observation was further supported by respondents who advanced reasons as to why the water sources were not being fully utilised. One Agritex officer in Ward 20 of Shurugwi district had this to say:

‘Water from Mutevekwi River is not being fully utilised by the farmers because most of the times, the irrigation pump engine for Ruchanyu irrigation scheme will always be down. This situation has led some farmers to rely on rain-fed agriculture, with only a few farmers being able to make use of their small irrigation pump engines to irrigate their crops.’ (Agritex Officer, Male, Shurugwi district)

Thus, several households allocated farming land in these schemes were said to be vulnerable to food insecurity because of lack of capacity to fully utilise the irrigable land. Most of the interviewees believed the irrigation schemes need to be rehabilitated so that farmers can be able to boost food production and improve household food security. This would ultimately help also in reducing poverty. The findings of this study agree with the results of a study by Mutiro and Lautze (2015), whose findings were that the improvement of agriculture through smallholder irrigation schemes helps in improving the livelihoods of the rural communities and reducing poverty. The results also support the view of SDGs framework, with SDG 2 (end hunger, achieve food security and improved nutrition and promote sustainable agriculture) encompassing targets for agricultural productivity, production and farmers’ income, and maintenance of ecosystems, agricultural markets and trade rules (Council of the European Union 2017).

### Initiatives towards the revival of irrigation schemes

The respondents revealed that several players are involved in the development and rehabilitation of small-scale irrigation schemes in Shurugwi district. It was gathered from the respondents that organisations that include Todal Mining Company, Unki Mining Company, Tongogara Community Share Ownership Trust (CSOT), Tongogara Rural District Council and government departments, including Agritex, were amongst the key players that had aided to rehabilitate the irrigation schemes in the district. The respondents reiterated that the assistance offered was in the form of farming machinery and irrigation equipment. The Department of Agritex provided skills training and knowledge on how to use some of the irrigation equipment. Information received from the Shurugwi district Administrator’s office indicates that the Tongogara CSOT normally assisted with funds. The following narration from one interviewee illustrates the types of assistance rendered:

‘Ruchanyu irrigation scheme once received funds for repairing the broken-down water pump engine. In addition, the same irrigation scheme also received irrigation equipment that encompasses irrigation pipes and fencing material. The electricity transformer was restored at the initiation of the European Union. However, there are still some problems since there is constant breakdown of the equipment and electricity power outages.’ (Male, smallholder farmer, Ruchanyu irrigation, Shurugwi)

Similarly, the researchers learnt that Gutsaruzhinji irrigation had also received some fencing material from Unki Mining Company to fence off their farming area from cattle and other wild animals that can destroy their crops. This move is seen as a step in the right direction as it ensures safe crop production and, resultantly, improved food security. As a way of developing the smallholder irrigation schemes in the district, Todal Mining Company has helped the smallholder farmers for Gutsaruzhinji scheme through tilling their land using its own tractors for free. These findings concur with the study by Moyo (2016) in Matabeleland North, who found that development partners such as the Catholic Agencies for Overseas Development (Cafod), Environment Africa and Caritas were instrumental in the rehabilitation of small irrigation schemes in the province. Moyo (2016) further argues that several irrigation schemes in Matabeleland North province managed to transform their operations through adopting climate-smart irrigation techniques. Solar power was installed to pump water, canals were repaired, and brick reservoirs were built by development partners. The findings are also in line with the framework of the study, as improve food security has a potential to end hunger and eradicate poverty.

### Benefits that can accrue to smallholder farmers from small-scale irrigation schemes

The respondents had the opinion that most smallholder farmers practising small-scale irrigation farming in the district had the potential to produce a variety of crops such as maize, beans, tomatoes, butternuts and green vegetables. Such a situation is likely to improve the food security of the farmers, as food would be readily available and accessible. Food availability and accessibility are some of the key components of food security, together with food utilisation and stability of food supply. Furthermore, the interviewees thought that the farmers could raise constant income from producing and marketing their produces. Thus, with small-scale irrigation schemes, the respondents opined that the farmers in the district are now able to grow their crops all year round, contrary to the period before the schemes were established where the farmers would rely on erratic rains. The growing of crops throughout the year would address the dimension of ‘stability of food supply’, thereby ensuring that the farmers are food secure because they would access food on a continuous basis. These findings also support the study by Muzerengi and Mapuranga (2017) that small-scale irrigation schemes in Zimbabwe can enable farmers to diversify production to new types of marketable crops, including a variety of cash crops and vegetables. This shows that, with the development and rehabilitation of small-scale irrigation schemes, most rural farmers in the district can improve their standard of living through the selling of agricultural produce (Mhembwe & Dube 2017). As such, small-scale irrigation schemes can be interpreted to play an important role in the development of a cash economy for many rural communities, with income becoming accessible to many individuals. It is safe to emphasise that small-scale irrigation schemes have a potential to improve food security of many communities.

Data from some of the farmers show that some households were now able to produce enough to feed themselves, an improvement from the situation when the farmers were relying solely on rain-fed agriculture. However, according to the farmers, the use of irrigation enabled them to produce surpluses such that even the poor had access to the food because of its abundance in the community. This is in line with the findings of Nhundu and Mushunje (2012) that irrigation farming can increase household food security in areas with poor rainfall, not only for the farmers but also for the rest of the community. Furthermore, the findings concur with SDG1, which emphasises that agriculture is a potent force for poverty reduction, especially in many developing countries (OECD 2016). The authors, therefore, contend that many farmers can realise some trickle-down effects because of embarking on irrigation farming. It is also the authors’ strong contention that because of small-scale irrigation farming, farmers can transform themselves from a state of helplessness to improved food security through generating more income from farming. Hence, Nhundu and Mushunje (2012) assert that if rural farmers have access to irrigation, they would also have the potential to contribute to poverty reduction and transform the lives of people.

### Challenges faced by smallholder farmers in running small-scale irrigation schemes in rural areas

In as much as the study managed to establish some of the benefits that the smallholder farmers are accruing through the adoption of the small-scale irrigation schemes, the researchers further learnt that several challenges exist to the farmers. According to respondents’ narrations, Ruchanyu irrigation scheme has been operating for a long time with malfunctioning water pump engine. The farmers indicated that they had no capacity and expertise to either fix the water pump engine or to replace it with a new one. As for Gutsaruzhinji irrigation scheme, the respondents revealed that some farmers were relying more on small individual water pumps that use fuel to irrigate their crops as the electricity power had been disconnected because of non-payment of electricity bills. It is the authors’ view that the farmers using individual fuel water pumps are likely to have their land either producing little or lying idle in the absence of enough rains. However, it was established from the farmers that efforts were under way to get electricity restored on the scheme, and that the recently acquired modern irrigation equipment under ‘More food for Africa’ programme was still lying idle because of the unavailability of electricity. The ‘More Food for Africa’ programme was a result of a bilateral agreement between the Government of Zimbabwe and the Government of Brazil. The agreement saw the latter supplying machinery and equipment to Zimbabwe to help boost agricultural production in the country.

One other challenge faced by the smallholder farmers was that of lack of enough farm machinery, in the form of tractors to plough the land. In addition, most farmers indicated that they lacked the technical skills to repair or service some of the machinery acquired under the ‘More food for Africa’ government programme. Through the observation method, the researchers noticed that Ruchanyu irrigation scheme had a newly acquired tractor grounded because of lack of technical skills amongst the farmers to service and repair their machinery. The following is a narration from an Agritex officer for ward 20:

‘Other contributing factors why the farmers are failing to repair the tractor is their lack of necessary skills, lack of cooperation among themselves, as well as lack of sense of ownership of the irrigation machinery and equipment.’ (Male, Agritex officer, Shurugwi district)

Another challenge faced by farmers, according to the respondents, was the payment of exorbitant water bills charged by the ZINWA, which is a government parastatal. Paying ZINWA for the supply of water drawn from the nearby water bodies (dams and rivers) was a burden to most smallholder farmers running small-scale schemes in the district. Thus, the farmers felt that high water charges by ZINWA were an extra burden to the already other existing challenges they faced. To further compound their challenges, the researchers observed that most of the farmers involved in the irrigation schemes were the elderly people. These had little capacity to actively participate in the farming activities to fully utilise the land. In addition, the elderly farmers were finding it difficult to easily adapt to the modern farming practices encouraged by the officers from the Department of Agritex to increase on productivity. The authors view this as contrary to the studies by Gebrehiwot et al. (2015), who argue that the elderly people have relatively richer experience of farming activities because they have better access to land than younger people.

## Conclusion and recommendations

The small-scale irrigation schemes are normally located close to water bodies where it is easier and cheap for the rural farmers to draw water for irrigation purposes. However, several of these schemes are being underutilised with machinery being vandalised, a move that can further comprise the food security situation of fragile communities. Governments of mainly less developed countries together with other stakeholders made several efforts to promote food security for the vulnerable rural communities particularly in low lying areas where rainfall is always erratic through the establishment and rehabilitation of small-scale irrigation schemes, but this has been found to be in vain because of the many challenges such rural farmers faced. The move to promote food security of the vulnerable communities is an effort to address the SDGs. This is particularly relevant to SDG1 and SDG2 that aim to end extreme poverty in all forms by 2030 and to end hunger, achieve food security and improved nutrition and promote sustainable, respectively. As such, the study maintains that the revival of small-scale rural irrigation schemes remains critical in promoting food security in arid and semi-arid regions of the world despite the numerous obstacles that can impede the processes. This study, however, recommends that farmers for small-scale irrigation schemes should be empowered with technical skills related to irrigation farming so that they can be able to overcome some of the challenges they face when it comes to the repairing and maintenance of their irrigation equipment. The study further recommends that stakeholders assist the farmers by putting in place infrastructure, including water reservoirs from which farmers can draw water in the event of water pump engines breaking down. Lastly, the study also recommends that the small-scale rural farmers need to be assisted in installing solar water pumps for irrigation purposes to minimise disruptions on their operations because of power outages. Such a move is also likely to assist small-scale farmers in cutting on electricity bills that are a bit on the expensive side in most African countries. These recommendations have a potential of promoting food security for vulnerable communities, thereby achieving the aim of the SDGs. This work has a potential to benefit smallholder farmers and other members of communities in the less developed world.
